# Synthesis, X-ray Single Crystal Structure, Molecular Docking and DFT Computations on *N*-[(1*E*)-1-(2*H*-1,3-Benzodioxol-5-yl)-3-(1*H*-imidazol-1-yl)propylidene]-hydroxylamine: A New Potential Antifungal Agent Precursor

**DOI:** 10.3390/molecules22030373

**Published:** 2017-02-28

**Authors:** Reem I. Al-Wabli, Alwah R. Al-Ghamdi, Hazem A. Ghabbour, Mohamed H. Al-Agamy, James Clemy Monicka, Issac Hubert Joe, Mohamed I. Attia

**Affiliations:** 1 Department of Pharmaceutical Chemistry, College of Pharmacy, King Saud University, P.O. Box 2457, Riyadh 11451, Saudi Arabia; ralwabli@KSU.EDU.SA (R.I.A.-W.); toto24se@hotmail.com (A.R.A.-G.); ghabbourh@yahoo.com (H.A.G.); 2 Department of Medicinal Chemistry, Faculty of Pharmacy, Mansoura University, Mansoura 35516, Egypt; 3 Department of Pharmaceutics, College of Pharmacy, King Saud University, P.O. Box 2457, Riyadh 11451, Saudi Arabia; malagamy@ksu.edu.sa; 4 Microbiology and Immunology Department, Faculty of Pharmacy, Al-Azhar University, Cairo 11884, Egypt; 5 Muslim Arts College, Thiruvithancode 629174, Tamil Nadu, India; clemymonicka@gmail.com; 6 Centre for Molecular and Biophysics Research, Mar Ivanios College, Thiruvananthapuram 695015, Kerala, India; hubertjoe@gmail.com; 7 Medicinal and Pharmaceutical Chemistry Department, Pharmaceutical and Drug Industries Research Division, National Research Centre (ID: 60014618), El Bohooth Street, Dokki, Giza 12622, Egypt

**Keywords:** crystal structure, imidazole, benzodioxole, oxime, DFT

## Abstract

Mycoses are serious health problem, especially in immunocompromised individuals. A new imidazole-bearing compound containing an oxime functionality was synthesized and characterized with different spectroscopic techniques to be used for the preparation of new antifungal agents. The stereochemistry of the oxime double bond was unequivocally determined via the single crystal X-ray technique. The title compound **4**, C_13_H_13_N_3_O_3_·C_3_H_8_O, crystallizes in the monoclinic space group *P*2_1_with *a* = 9.0963(3) Å, *b* = 14.7244(6) Å, *c* = 10.7035(4) Å, β = 94.298 (3)°, *V* = 1429.57(9) Å^3^, *Z* = 2. The molecules were packed in the crystal structure by eight intermolecular hydrogen bond interactions. A comprehensive spectral analysis of the title molecule **4** has been performed based on the scaled quantum mechanical (SQM) force field obtained by density-functional theory (DFT) calculations. A molecular docking study illustrated the binding mode of the title compound **4** into its target protein. The preliminary antifungal activity of the title compound **4** was determined using a broth microdilution assay.

## 1. Introduction

The incidence of systemic fungal infections (mycoses) has increased drastically in recent years, mainly in immunosuppressed or immunocompromised individuals with AIDS, cancer or undergoing organ transplantation [1,2]. Failure of the available antifungal agents to treat fungal infections is primarily due to dramatic increase in resistance to the conventional antifungal drugs leading to morbidity and mortality in patients facing life-threatening fungal infections. In order to overcome this serious problem, the development of new alternative antifungal drug therapies with improved efficacy, broader activity and favorable safety profile has attracted a great deal of interest [3,4].

Azole-based compounds having either an imidazole or triazole pharmacophore moiety in their structure, constitute the mainstay of the antifungal chemotherapeutic agents used in the clinic [5,6]. Azoles competitively inhibit cytochrome P450-dependent lanosterol 14α-demethylase (CYP51) resulting in depletion of ergosterol in fungi making them unable to grow in a normal way [7,8]. A screening the literature revealed that most of the available imidazole-bearing antifungal agents have two carbon spacers between the imidazole moiety and an aromatic residue, while few antifungals have a three carbon spacer between the pharmacophore and the aromatic part [9,10,11].

On the other hand, the benzodioxole moiety is found in a sizable number of biologically active compounds with a wide range of activities [12,13,14,15,16]. The title molecule features both the 1,3-benzodioxole moiety and the imidazole nucleus connecting to each other through a three carbon bridge. Therefore, the current investigation deals with the synthesis, molecular characterization and single crystal X-ray structure of a new oxime derivative, namely *N*-[(1*E*)-1-(2*H*-1,3-benzodioxol-5-yl)-3-(1*H*-imidazol-1-yl)propylidene]hydroxylamine (**4**) to be utilized as a potential precursor for imidazole-bearing antifungal agents. The stereochemistry of the imine functionality in the title molecule **4** was certainly determined via the single crystal X-ray crystallography technique. In addition, density-functional theory (DFT) computations were also performed as a useful tool to investigate the electronic structure and molecular geometry of the title molecule **4**. Molecular docking studies were conducted in order to predict the biological activity of compound **4**.

## 2. Results and Discussion

### 2.1. Chemistry

**Scheme 1:**
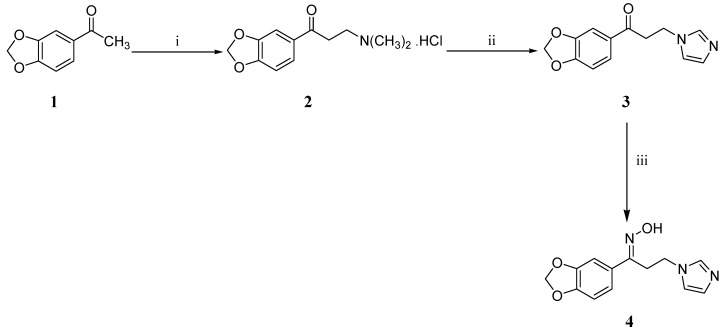
Synthesis of the target oxime **4**. *Reagents and conditions*: (i) HN(CH_3_)_2_.HCl, (CH_2_O)*_n_*, conc. HCl, ethanol, reflux, 2 h; (ii) Imidazole, water, reflux, 5 h; (iii) H_2_NOH.HCl, KOH, ethanol, reflux, 18h.

Scheme 1 illustrates the synthetic pathway which was adopted to synthesize the target compound **4**. The synthesis commenced with a Mannich reaction under acidic conditions using the commercially available 1-(2*H*-1,3-benzodioxol-5-yl)ethanone (**1**). Subsequently, the formed Mannich base hydrochloride **2** was smoothly transformed in aqueous solution into the ketone **3** via a nucleophilic substitution reaction using imidazole. The target oxime **4** was ultimately obtained using the standard procedure for oxime formation with hydroxylamine hydrochloride in ethanol in the presence of potassium hydroxide [17].

### 2.2. Crystal Structure of the Title Compound **4**

**Figure 1:**
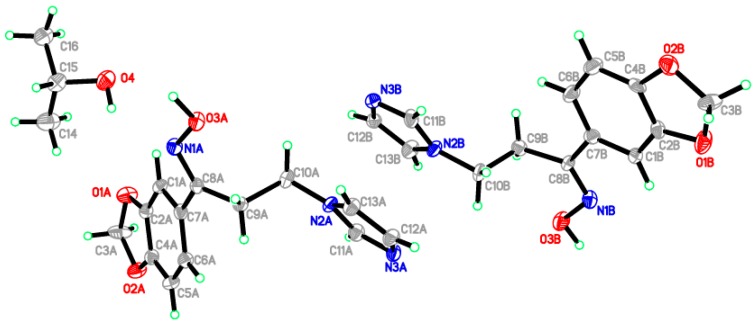
ORTEP diagram of the title compound drawn at 40% ellipsoids for non-hydrogen atoms showing two molecules and one isopropanol molecule as a solvent.

**Figure 2:**
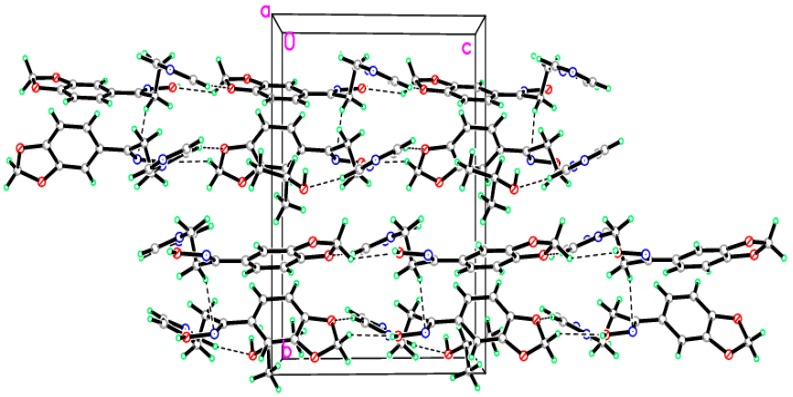
Crystal packing showing intermolecular hydrogen bonds as dashed lines along the *b* and *c* axes.

The configuration of the target compound **4** was confirmed via X-ray crystallography. A suitable single colorless crystal of dimensions, 0.40 × 0.23 × 0.11 mm, was selected for X-ray diffraction analysis. The labeled displacement ellipsoid plot of this molecule is shown in Figure 1. Figure 2 depicts the packing of the molecules in the crystal structure.

The single crystal X-ray molecular structure therefore conformed the assigned (*E*)-configuration of the imine group in the target compound. The crystal structure contains two independent molecules with one isopropanol molecule as a solvent in the asymmetric unit. The benzodioxole ring (C1/C2/O1/C3/O2/C4–C7) forms dihedral angles of 44.04(3)° and 20.04(2)° with the imidazole ring (N2/C11/N3/C12/C13) for molecules A and B, respectively. The crystal structure is stabilized by eight hydrogen bonds along the *b* and *c*-axis (Table 1).

### 2.3. Structural Geometry Analysis

**Figure 3:**
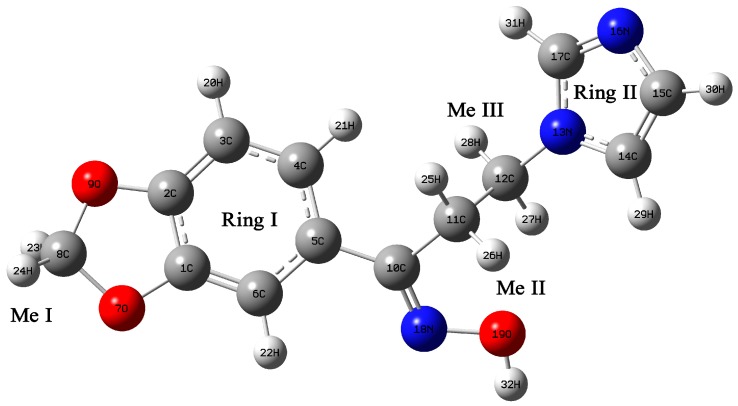
Optimized molecular structure of compound **4**.

Equilibrium structural geometry of the title molecule **4** has been evaluated by a potential energy surface (PES) scan study. The flexible dihedral angles of C_11_–C_12_–N_13_–C_14_, C_11_–C_10_–C_5_–C_4_ and C_11_–C_10_–N_18_–O_19_ were scanned from 0° to 360° and their optimum energy was studied to identify stable conformations of the title compound **4**. The global minimum energy is –893.45 and –893.49 Hartree for the conformers of compound **4** in the gas and solution phases, respectively (various conformers of compound **4** are shown in Figure S1). From this PES analysis, we have identified the minimum energy conformer of this molecule which was chosen for the subsequent studies. The optimized structure of the studied compound **4** with atoms numbering is depicted in Figure 3. Optimized bond lengths, bond angles and dihedral angles have been presented in Table 2. The formation of intramolecular hydrogen bonding is exposed by the intramolecular contacts to H_22_···N_18_ occur with H···O distance of 2.455 Å, which is shorter than the van der Waals separation between the O and H atoms (2.75 Å) [18]. Shortening of the C–N bond lengths N_13_–C_14_, N_13_–C_17_, N_16_–C_17_ and N_16_–C_15_ is typical for double bonds and it is due to resonance interactions. The linear fitting graphs (Figure S2) are drawn to study the correlation between the average experimental values and computed results. Statistical analysis revealed that the results of the computed solvation model are in a good agreement with the average experimental XRD values. Therefore, this method was considered to compute spectral vibrations, natural bond orbital and Frontier orbital energy analyses for the title molecule **4**.

### 2.4. Natural Bond Orbital Analysis

Natural bond orbital (NBO) analysis describes the (hyper)conjugative interactions between donor–acceptor orbitals in order to understand intramolecular charge–transfer phenomenon of the molecular system [19]. Selective donor–acceptor interactions of the oxime **4** are listed along with their occupancy and stabilization energy values in Table 3. The hyperconjugative interactions of π(C_1_–C_6_)→π*(C_2_–C_3_), π(C_1_–C_6_)→π*(C_4_–C_5_), π(C_2_–C_3_)→π*(C_1_–C_6_), π(C_2_–C_3_)→π*(C_4_–C_5_), π(C_4_–C_5_)→π*(C_1_–C_6_), and π(C_4_–C_5_)→π*(C_2_–C_3_) were 20.27, 17.86, 18.94, 19.07, 17.00 and 17.47 kcal/mol, respectively. This could be attributed to the charge delocalization leading to ring resonance effect. The lone pair conjugative interactions of LP(2)O_7_→π*(C_1_–C_6_), LP(2)O_9_→π*(C_2_–C_3_), LP1(N_13_)→π*(C_14_–C_15_), and LP1(N_13_)→π*(N_16_–C_17_) have stabilization energy of 25.80, 27.07, 30.67, and 46.28 kcal/mol, respectively. The large E(2) values revealed the occurrence of strong electron delocalization over the ring moieties. These interactions were observed as an increase in the electron density (ED) of the C–C antibonding orbital, which weakens the respective bonds. The C_10_=N_18_ bond length (1.2861 Å) is significantly shorter than the other –CN bonds and the electron density (ED) of this antibonding orbital was decreased to 0.18707, which is an evidence for the rehybridization [20]. More conjugative and hyperconjugative interactions were formed in the lone-pair, C–C and C–C bond orbital overlap which confirms the intramolecular charge-transfer (ICT) causing the stabilization of the molecular structure of the title oxime **4**.

### 2.5. Vibrational Analysis

**Figure 4:**
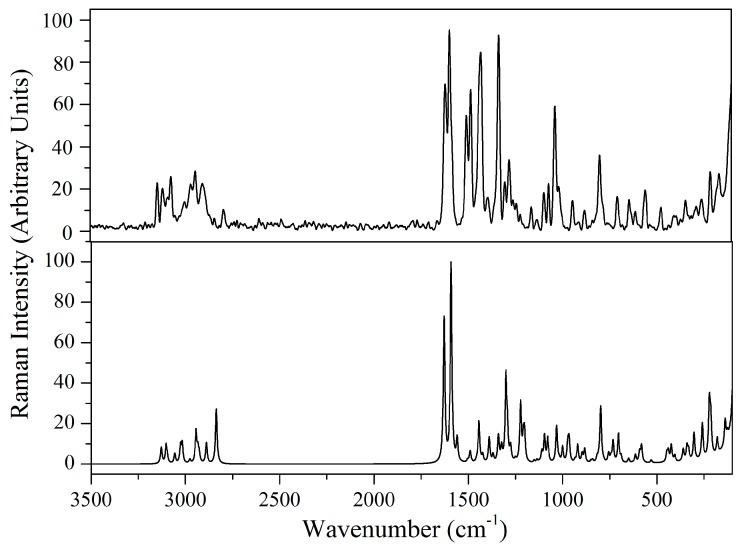
Experimental (**upper**) and simulated (**lower**) FT-Raman spectra of compound **4**.

**Figure 5:**
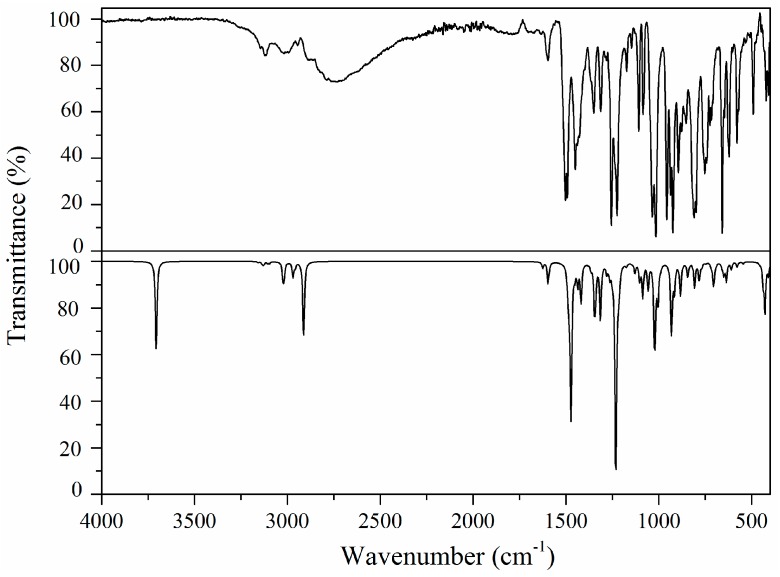
Experimental (**upper**) and simulated **(lower**) FT-IR spectra of compound **4**.

The title molecule **4** consists of 32 atoms and their characteristic vibrations are described by 90 normal modes. FT-Raman and FT-IR spectra of the title compound **4** are shown in Figure 4 and Figure 5, respectively. Theoretical and experimental spectral data of the target oxime **4** are presented in Table 4, including calculated and fundamental wavenumbers, FT-IR- and FT-Raman intensities along with the tentative vibrational assignment. The calculated vibrational wavenumbers were corrected by scaled quantum mechanical force-field (SQMFF) method [21] by selective scaling factor approach [22]. Using this force-field method, the calculated wavenumbers are reproducing the experimental values with a mean deviation of 15 cm^–1^. Internal valence coordinates and scaling factors information of the title compound **4** modes are given in Tables S1 and S2, respectively.

#### 2.5.1. Imidazole Ring Vibrations

The calculated IR and Raman imidazole C–H stretching vibrations at 3118 and 3145 cm^–1^ are in close agreement with the experimental wavenumbers observed at 3144, 3125, 3120 cm^–1^ [23,24]. The FT-IR bands observed at 1503, 1350, 1280 and 1108 cm^–1^ and the FT-Raman ones at 1516, 1346, 1291, 1107 and 1050 cm^–1^ are definitely attributed to imidazole CH in-plane bend modes which are coupled with several other modes. The FT-IR bands at 752 and 725 cm^–1^ and the weak FT-Raman bands at 721 cm^–1^, can be assigned to the out-of-plane C–H wagging modes, as supported by theliterature data [25]. The C_14_=C_15_ stretching mode is the most characteristic vibration of the heterocyclic imidazole ring which was observed as medium intensity bands at 1493 (FT-IR) and at 1494 (FT-Raman) cm^–1^. The FT-IR and FT-Raman vibrations of the imidazole ring out-of-plane torsion modes appeared at 658, 578 (FT-IR) and 573 (FT-Raman) cm^–1^.

#### 2.5.2. Methylene Group Vibrations

The neighboring rings π-system and nitrogen lone pair affects the spectral behavior of *sp*^3^ hybridized methylene moiety. Methylene asymmetric and symmetric C–H stretching vibrations usually appear in the region 2960–2840 cm^–1^ [26]. The asymmetric (2970 and 2946 cm^–1^) and symmetric (2908 and 2845 cm^–1^) stretching contributing bands appeared as a medium intensity band in the FT-Raman spectrum of the title compound **4**. Lowering of symmetric stretching wavenumbers could be attributed to the hyperconjugative interaction between the nitrogen lone pair and σ*(C–H) bond. The rocking, wagging and twisting vibrational modes of compound **4** appeared in the region of 1400–900 cm^–1^ [27]. The CH_2_ scissoring mode appeared as a characteristic band near 1450 and 1439 cm^–1^ in the FT-IR spectrum and at 1440 cm^–1^ in the FT-Raman spectrum of the title oxime **4**. The normal coordinate analysis (NCA) supports the FT-Raman bands at 1254 and 1271 cm^–1^ and the FT-IR bands at 1255 cm^–1^ for the unambiguous assignment of CH_2_ twisting and wagging modes.

#### 2.5.3. Benzodioxole Ring Vibrations

Aromatic C–H stretching mode appears in the region of 3000–3100 cm^–1^. Weak FT-Raman bands identified at 3073 and 3001 cm^–1^ have been assigned to the C–H stretching mode. Usually the bands due to ring C–H in-plane and out-of-plane bending vibrations are observed in the region of 1000–1300 and 750–1000 cm^–1^, respectively. In the title compound **4**, the C–H in-plane bending vibration has been observed as a medium intensity FT-IR band at 1313 cm^–1^ and as a weak band at 1314 cm^–1^ in the FT-Raman spectrum. The medium to weak intensity bands observed at 854 (FT-IR), 850 (FT-Raman), 814 (FT-Raman) and 808 (FT-IR) cm^–1^ have been assigned to C–H out-of-plane bending mode. Ring C–C stretching modes have been identified in the FT-IR spectrum of the target compound **4** at 1595, 1428 and 1173 cm^–1^ while they appeared at 1606 and 1174 cm^–1^ in the FT-Raman spectrum. The ring asymmetric deformation and torsion modes have been identified at 716, 556, 492 and 417 cm^–1^ in the FT-IR and at 490 cm^–1^ in the FT-Raman spectra of the title molecule **4**.

### 2.6. Frontier Molecular Orbital Analysis

**Figure 6:**
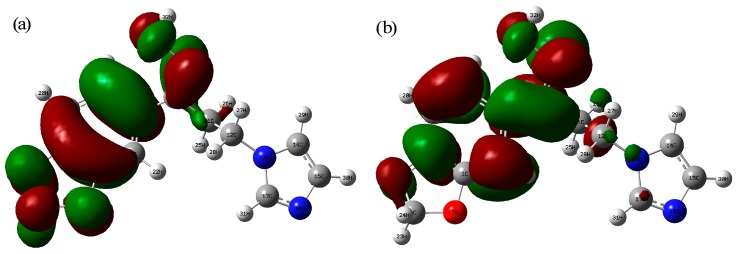
(**a**) HOMO (**b**) LUMO orbital of compound **4**.

A detailed knowledge of molecular electron density distribution and electron motion are necessary to understand molecular recognition and chemical reactivity of the molecule. The LUMO and HOMO orbital energy analysis of the title compound **4** has been computed using DFT method in the solution phase (isopropanol). The electron charge cloud is located at the piperonal ring in highest occupied molecular orbital (HOMO) while it is located mainly at the phenyl ring in the lowest unoccupied molecular orbital (LUMO). The energy of the HOMO is −6.23 eV and LUMO is −1.60 eV giving rise to a HOMO-LUMO energy gap of 4.63 eV. The HOMO-LUMO energy gap value supports the intramolecular charge-transfer interactions within the title molecule. The HOMO and LUMO orbital diagram is shown in Figure 6.

### 2.7. NMR Chemical Shift Analysis

The carbon and hydrogen chemical shift values of the title oxime **4** were calculated based on Gauge-independent atomic orbital (GIAO) method at B3LYP/6-311++G(d,p) level of theory [28]. The computed values are presented in Table 5. The correlation graphs were plotted over the observed and predicted chemical shift values of the title molecule **4** and the linear fitting plots are shown in Figure S3.

The phenyl carbons signals usually appear in the region of 120–140 ppm. The C1 and C2 in the title molecule **4** were observed at 148.0 and 148.3 ppm, respectively. This downfield chemical shift values revealed that, these carbon atoms bounded with the electronegative oxygen atoms. In general, imidazole ring protons signals occur in the region of 6–8 ppm. The ^1^H-NMR spectrum of the target oxime **4** showed signals at 6.89, 7.19 and 7.66 ppm which correspond to the protons of the imidazole ring. The aromatic piperonal ring protons were observed at 6.84 (as doublet), 7.06 (as doublet) and 7.13 (as singlet) ppm. There is a good agreement between the calculated and observed chemical shift values of the title molecule **4** with a correlation coefficient (*R*^2^) values = 0.994 and 0.953 for ^13^C and ^1^H, respectively.

### 2.8. Molecular Docking Study

**Figure 7:**
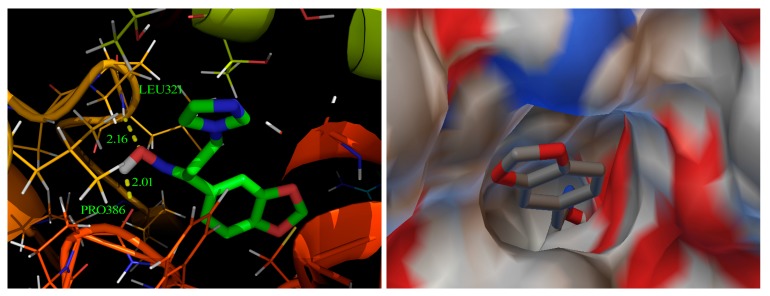
Binding pose of compound **4** with its target protein.

Molecular docking is an important technique to predict the biological activity of chemical compounds [29]. The target compound **4** was energy minimized using DFT method with the help of Gaussian program [30]. The target protein (cytochrome P450-dependent (CYP51) lanosterol 14α-demethylase enzyme) for antifungal azoles has been identified based on a multilevel neighborhoods of atoms’ (MNAs) algorithm model by the PASS online server [31]. This target protein (PDB code: 1EA1) was downloaded from the research collaboratory structural bioinformatics (RCSB) protein data bank [32]. The target compound **4** was docked using AutoDock Tools 1.5.4 (The Scripps Research Institute, La Jolla, CA, USA) interfaced with the AutoDock 4.2 program in the rigid docking methodology [33,34]. The binding free energy and inhibition constant of the proper conformation of the title compound **4** was predicted to be −5.06 kcal/mol and 195.75 μM, respectively. The hydrogen bond interaction of protein-ligand complex is shown in Figure 7. The amino acid residues LEU321 and PRO386 of the target protein are bounded with the title compound **4** by an intermolecular hydrogen bonding interaction. The docking study illustrated the affinity of compound **4** toward its target protein with a good binding energy value (–5.06 kcal/mol) and hence its suitability as a potential precursor to prepare new antifungal agents.

### 2.9. Antifungal Activity of the Title Compound ***4***

Table 6 presents the preliminary antifungal activity of the tested oxime **4** as well as the reference standard drug, ketoconazole. Compound **4** exhibited a MIC value of 987.43 μmol/L against *C. albicans*, *C. tropicalis*, *C. parapsilosis* and *Aspergillus niger* in the broth microdilution assay. While inactive itself, taken together the body of evidence suggests that compound **4** could be used as a starting material to prepare new antifungal agents with better antifungal profile.

## 3. Experimental

### 3.1. General

Melting points were measured using a Gallenkamp melting point device, and are uncorrected. Infrared (IR) spectra (as KBr disks) were recorded on FT-IR Spectrum BX device (Perkin Elmer, Ayer Rajah Crescent, Singapore). The NMR samples were dissolved in either CDCl_3_ or DMSO-*d*_6_ and the NMR spectra were recorded using a Bruker NMR spectrometer (Bruker, Reinstetten, Germany), at 500 MHz for ^1^H and 125.76 MHz for ^13^C at the Research Center, College of Pharmacy, King Saud University, Saudi Arabia. Chemical shifts are expressed in δ-values (ppm) relative to TMS as an internal standard. Mass spectra were recorded using a Quadrupole 6120 LC/MS equipped with an electrospray ionization (ESI) source (Agilent Technologies, Palo Alto, CA, USA). Silica gel TLC (thin layer chromatography) plates (silica gel precoated aluminium cards with 254 nm fluorescent indicator) from Merck (Darmstadt, Germany) were used for thin layer chromatography. Visualization was performed by illumination with a UV light source (254 nm).

### 3.2. Synthesis

#### 3.2.1. Synthesis of 1-(2*H*-1,3-Benzodioxol-5-yl)-3-(1*H*-imidazol-1-yl)propan-1-one (**3**)

A catalytic amount of concentrated hydrochloric acid (0.5 mL) was added to a mixture containing paraformaldehyde (0.81 g, 9.0 mmol), dimethylamine hydrochloride (2.20 g, 27 mmol) and1-(2*H*-1,3-benzodioxol-5-yl)ethanone (**1**, 3.28 g, 20 mmol) in absolute ethanol (15 mL). The reaction mixture was refluxed for two hours, cooled and acetone (30 mL) was added to precipitate the Mannich base hydrochloride (**2**) which was collected by filtration and dried. Imidazole (1.36 g, 20 mmol) was added to a solution of compound **2** (2.58 g, 10 mmol) in water (10 mL) and the solution was refluxed for five hours, cooled and the precipitated solid was filtered off to afford compound **3**. Compound **3** was re-crystallized from ethanol to give 1.15 g (47%) of the pure pivotal ketone **3** as a white solid m.p. 150–152 °C. IR (KBr): ν (cm^–1^) 3115, 2968, 1757 (C=O), 1637 (C=N), 1600, 1494, 1255, 750; ^1^H-NMR (CDCl_3_): δ (ppm) 3.37 (t, *J* = 6.5 Hz, 2H, –C*H_2_*–CH_2_–N), 4.42 (t, *J* = 6.5 Hz, 2H, –CH_2_–C*H_2_*–N), 6.05 (s, 2H, –O–C*H_2_*–O–), 6.84 (d, *J* = 8.0 Hz, 1H, Ar–H), 6.99 (s, 1H, –N–C*H*=CH–N=), 7.04 (s, 1H, –N–CH=C*H*–N=), 7.39 (d, *J* = 1.5 Hz, 1H, Ar–H), 7.50 (dd, *J* = 1.5, 8.0 Hz, 1H, Ar–H), 7.64 (s, 1H, –N–C*H*=N–); ^13^C-NMR (CDCl_3_): δ (ppm) 39.6 (–*C*H_2_–CH_2_–N), 41.7 (–CH_2_–*C*H_2_–N), 102.0 (–O–*C*H_2_–O–), 107.7 (Ar–CH), 108.0 (Ar–CH), 119.2 (–N–*C*H=CH–N=), 124.4, 129.1 (Ar–CH, –N–CH=*C*H–N=), 131.0, (Ar–C), 137.4 (–N–*C*H=N–), 148.4, 152.3 (Ar–C), 194.6 (C=O); MS *m*/*z* (ESI): 245.0 [M + H]^+^.

#### 3.2.2. Synthesis of (1*E*)-1-(2*H*-1,3-Benzodioxol-5-yl)-*N*-hydroxy-3-(1*H*-imidazol-1-yl)propan-1-imine (**4**)

Potassium hydroxide (1.12 g, 20 mmol) was added to a mixture containing hydroxylamine hydrochloride (1.39 g, 20 mmol), ketone **3** (2.44 g, 10 mmol) in ethanol (10 mL). The reaction mixture was refluxed under stirring for 18 hours, cooled to room temperature and the insoluble matter was collected by filtration. The filtrate was concentrated under reduced pressure and the residue was poured onto ice-cold water (15 mL). The obtained solid was filtered off and dried to yield 1.7 g (64%) of the target oxime **4** as a white solid m.p. 142–144 °C. Re-crystallization of the oxime **4** from isopropanol gave colorless single crystals which were suitable for X-ray analysis. ^1^H-NMR (DMSO-*d*_6_): δ(ppm) 3.14 (t, *J* = 7.0 Hz, 2H, –C*H_2_*–CH_2_–N), 4.17 (t, *J* = 7.0 Hz, 2H, –CH_2_–C*H_2_*–N), 6.04 (s, 2H, –O–C*H_2_*–O–), 6.84 (d, *J* = 7.5 Hz, 1H, Ar–H), 6.89 (s, 1H, –N–C*H*=CH–N=), 7.06 (dd, *J* = 1.5, 8.0 Hz, 1H, Ar–H), 7.13 (d, *J* = 1.0 Hz, 1H, Ar–H), 7.19 (s, 1H, –N–CH=C*H*–N=), 7.66 (s, 1H, –N–C*H*=N–), 11.41 (s, 1H, OH); ^13^C-NMR (DMSO–*d_6_*): δ(ppm) 28.2 (–*C*H_2_–CH_2_–N), 43.3 (–CH_2_–*C*H_2_–N), 101.7 (–O–*C*H_2_–O–), 106.1, 108.5 (Ar–CH), 119.9 (–N–*C*H=CH–N=), 120.5, 128.3 (Ar–CH, –N–CH=*C*H–N=), 130.3 (Ar–C), 137.5 (–N–*C*H=N–), 148.0, 148.3 (Ar–C), 153.7 (C=N–OH); MS *m*/*z* (ESI): 260.1 [M + H]^+^.

### 3.3. Crystal Structure Determination

Slow evaporation of the alcoholic (isopropanol) solution of the title compound **4** furnished its colourless block single crystals. The X-ray diffraction measurement of the target oxime **4** was conducted on a SMART APEXII CCD diffractometer (Bruker, Karlsruhe, Germany) equipped with graphite monochromatic CuK\a radiation (λ = 1.54178 Å) at 296 (2) K. Cell refinement and data reduction were done by Bruker SAINT [35]. SHELXS-97 [36] was used to solve and refine the title structure. The final refinement of the crystal structure of the title oxime **4** was performed by full- matrix least-squares techniques with anisotropic thermal data for non hydrogen atoms on *F^2^*. All the hydrogen atoms were placed in the calculated positions and constrained to ride on their parent atoms. Multi-scan absorption correction was applied by the use of SADABS software [35]. The crystallographic data and refinement information are summarized in Table 7. Crystallographic data of compound **4** have been deposited with the Cambridge Crystallographic Data Center (supplementary publication number CCDC-1508986). Copies of the data may be obtained free of charge from the Director, CCDC, 12 Union Road, Cambridge, CB2 1EZ, UK (deposit@ccdc.cam.ac.uk).

### 3.4. FT-IR and FT-Raman Measurements

The FT-Raman spectrum of the oxime **4** was recorded in the spectral range of 3500–50 cm^–1^ using a Bruker RFS-27 FT-Raman spectrophotometer (Bruker, Billerica, MA, USA). The 1064 nm line of Nd:YAG laser operating at 100 mW power was used for excitation. The FT-IR spectrum of the oxime **4** was recorded with a spectral resolution of 2 cm^–1^ in the 4000–400 cm^–1^ range. Solid sample in KBr pellets was used in the FT-IR measurements.

### 3.5. Quantum Chemical Calculations

Optimized structural geometry and harmonic vibrational wavenumbers have been calculated at DFT/B3LYP/6-311++G(d,p) level of basis set in the gas phase. The polarizable continuum model (PCM) using the integral equation formalism (IEF) variant is the self-consistent reaction field (SCRF) to predict the structural parameters and vibrational wavenumbers of the title molecule **4** and isopropanol has defined as the solvent in implicit solvation model. This calculation has been performed in the presence of isopropanol by placing the title molecule **4** in a cavity within the solvent reaction field. Normal coordinate analysis (NCA) has been performed to obtain detailed explanation of the molecular motion relating to the normal modes using the MOLVIB program version 7.0 written by Sundius [37,38]. According to the scaled quantum mechanical force-field (SQMFF) procedure [21], selective scaling has been performed in the natural internal coordinate representation [22]. The simulated IR and Raman spectra of the title compound **4** have been plotted using pure Lorentizian band shapes with a bandwidth of full width height maximum of 10 cm^–1^. Second order interactions between the filled orbital of one subsystem and vacant orbitals of another subsystem have been understood with the aid of natural bonding orbitals (NBO) analysis [19] using NBO 3.1 program [39] as implemented in the Gaussian ‘09 package [30] at the DFT/B3LYP level. Molecular docking analysis using AutoDock 4.2 program [33] predicted the antifungal activity of the title compound **4**.

### 3.6. Antifungal Activity

#### 3.6.1. Materials

The reference standard antifungal drug, ketoconazole, was purchased from Sigma-Aldrich Co. (St. Louis, MO, USA). Liquid RPMI 1640 medium supplemented with l-glutamine was obtained from Gibco-BRL, Life Technologies (Paisley, Scotland). Sabouraud Dextrose Agar (SDA) was obtained from Merck Co. (Darmstadt, Germany). Dimethyl sulfoxide (100%) was used to dissolve ketoconazole, and/or the tested compound **4** to give an initial concentration of 2048 mg/L.

#### 3.6.2. Organisms

The used fungal strains are *Candida albicans* (ATCC 90028), *Candida tropicalis* (ATCC 66029), *Candida* *parapsilosis* (ATCC 22019) and *Aspergillus niger* (ATCC 16404).

#### 3.6.3. Preparation of Fungal Inocula

The inocula of the standard mold *Aspergillus niger* strain have been prepared by removing the sporulated *A. niger* from the Sabouraud Dextrose agar slant with a microbiological loop and the spores have been suspended in 10 mL of sterile water. The suspension has been filtered through sterile gauze to remove hyphae. The resulting suspension of conidia has been vigorously mixed using a vortex. The suspension has been adjusted to 1 × 10^5^ CFU/mL using spectrophotometer. This fungal suspension has been diluted 1:5 with RPMI medium to obtain suspensions having 2× of the required final concentration. This conidial suspension had a final concentration of 1 × 10^4^ CFU/mL when mixed with the tested solution of compound **4**. On the other hand, the inocula of the standard yeast strains of *C. albicans*, *C. tropicalis* and *C. parapsilosis* have been prepared by suspending five representative colonies, obtained from 24 to 48 h culture on Sabauraud Dextrose agar medium, in sterile distilled water. The final inoculum concentration must be between 0.5 × 10^5^ and 2.5 × 10^5^ CFU/mL.

#### 3.6.4. Preparation of the Tested Compound Solution

Briefly, a twofold dilution series of the tested compound **4** has been prepared in a double strength RPMI 1640 culture medium. Ten serial dilutions were prepared to give concentrations ranged from 1024 mg/L to 2 mg/L.

#### 3.6.5. Antifungal Susceptibility Studies

Minimum Inhibitory Concentrations (MICs) have been determined by broth microdilution testing as described previously by EUCAST [40]. The experiment was carried out in duplicate. Briefly, one mL of RPMI 1640 medium from each of the bottle containing the corresponding concentration of the tested compound **4** has been transferred into sterile 7 mL Sterilin tubes (Thermo Fisher Scientific, Waltham, MA, USA). The RPMI 1640 medium containing 1024 mg/L of the tested compound **4** has been dispensed to tube 1, the medium containing 512 mg/L has been dispensed to tube 2, the medium containing 256 mg/L has been dispensed to tube 3 and so on to tube 10 for the medium containing 2 mg/L of the tested compound **4**. One mL of the medium has been dispensed in tubes 11 (positive control) and 12 (negative control). One mL of the diluted inoculum suspension has transferred to each tube except tube 12 to bring the tested compound **4** dilutions to the required final test concentrations. The tubes were incubated at 35 °C for 72 h. The MIC of the tested compound **4** was determined visually by recording the degree of growth inhibition in each tube.

## 4. Conclusions

The synthesis and spectroscopic characterization of *N*-[(1*E*)-1-(2*H*-1,3-benzodioxol-5-yl)-3-(1*H*-imidazol-1-yl)propylidene]hydroxylamine (**4**) as new antifungal precursor has been reported. Computational studies on the target oxime **4** revealed that the theoretical wavenumbers are in a fair agreement with the observed wavenumbers except those associated with H-bonding. CH_2_ symmetric stretching wavenumber is red-shifted due to the hyperconjugative interaction between the nitrogen lone pair and σ*(C–H) bond. Single crystal X-ray analysis of the target molecule **4** confirmed the (*E*)-configuration of its imine double bond. A molecular docking study predicted the binding mode of compound **4** into its target protein and hence its usefulness as a potential precursor for new imidazole-bearing antifungal agents featuring both benzodioxole and imidazole pharmacophore moieties.

## Figures and Tables

**Table 1:** Hydrogen-bond geometry (Å, °) of the title molecule **4**.

*D*–H···*A*	*D*–H	H···*A*	*D*···*A*	*D*–H···*A*
O3A–H3OA···N3A ^i^	1.00(5)	1.73(5)	2.722(5)	176(6)
O3B–H3OB···N3B ^ii^	0.98(6)	1.70(6)	2.672(5)	175(8)
O4–H1O4···N1A	0.91(7)	2.38(7)	3.127(6)	139(6)
C3A–H3AB···O3A ^iii^	0.9900	2.4900	3.128(6)	122.00
C3B–H3BB···O3B ^iv^	0.9900	2.3600	3.080(6)	129.00
C11A–H11A···O4 ^ii^	0.9500	2.2700	3.179(7)	159.00
C13A–H13A···O2A ^iv^	0.9500	2.5600	3.459(6)	158.00
C13B–H13B···O2B ^iii^	0.9500	2.5200	3.424(6)	160.00
C9B–H9BB···N1A ^v^	0.9900	2.5800	3.502(6)	154.00

Symmetry codes: (i) *x* − 1, *y*, *z*; (ii) *x* + 1, *y*, *z*; (iii) *x*, *y*, *z* − 1; (iv) *x*, *y*, *z* + 1; (v) −*x* + 1, *y* + 1/2, −*z* + 1.

**Table 2:** Optimized structural geometry parameters for compound **4**.

Bond Length (Å)				Bond Angle (°)				Dihedral Angle (°)			
Parameters	Calculated		Exp.	Parameters	Calculated		Exp.	Parameters	Calculated		Exp.
	Gas Phase	Solution Phase			Gas Phase	Solution Phase			Gas Phase	Solution Phase	
C1–C2	1.3947	1.3945	1.379	C1–C2–C3	121.40	121.44	121.60	C1–C2–C3–C4	–0.32	–0.29	1.20
C2–C3	1.3753	1.3764	1.362	C1–C2–O9	109.69	109.67	110.05	C1–C2–C3–H20	179.6	179.72	–179.12
C3–C4	1.4049	1.405	1.395	C1–C6–C5	117.55	117.55	117.05	C1–C2–O9–C8	10.37	10.84	–4.10
C4–C5	1.4001	1.4005	1.394	C1–C6–H22	121.74	121.53	121.62	C1–O7–C8–O9	16.91	17.41	–4.55
C5–C6	1.4181	1.4183	1.411	C1–O7–C8	105.45	105.50	105.41	C1–O7–C8–H23	135.54	135.89	115.78
C6–C1	1.3707	1.3719	1.370	C2–C1–C6	122.44	122.37	122.65	C2–C1–O7–C8	–10.64	–10.95	1.95
C1–O7	1.3743	1.374	1.376	C2–C1–O7	109.26	109.35	109.85	C2–C3–C4–C5	0.39	0.51	–1.00
C2–O9	1.3695	1.3695	1.382	C2–C3–C4	116.93	116.98	117.25	C2–C3–C4–H21	–179.8	–179.96	178.51
C3–H20	1.0821	1.0822	0.950	C2–O9–C8	105.31	105.44	105.57	C2–C1–C6–C5	0.21	0.09	1.80
C4–H21	1.0814	1.0815	0.950	C3–C2–O9	128.89	128.87	128.35	C2–C1–C6–H22	–179.59	179.94	–179.81
C5–C10	1.4846	1.4847	1.482	C2–C3–H20	121.55	121.72	121.41	C3–C4–C5–C6	–0.17	–0.44	1.05
C6–H22	1.0805	1.0808	0.950	C4–C3–H20	121.52	121.30	121.44	C3–C2–C1–O7	–178.62	–178.62	179.60
C8–O7	1.4317	1.4372	1.424	C3–C4–C5	122.28	122.18	121.95	C3–C4–C5–C10	178.73	178.73	177.75
C8–O9	1.4360	1.4401	1.418	C3–C4–H21	117.57	117.55	119.03	C3–C2–O9–C8	–170.62	–170.62	177.8
C8–H23	1.0892	1.0877	0.990	C5–C4–H21	120.15	120.27	119.03	C4–C5–C6–C1	0.13	0.13	2.30
C8–H24	1.0966	1.0943	0.990	C4–C5–C6	119.38	119.48	119.62	C4–C5–C6–H22	–179.72	–179.72	179.78
C10–C11	1.5127	1.5133	1.519	C4–C5–C10	121.01	120.74	121.35	C4–C5–C10–C11	16.37	16.37	19.05
C10–N18	1.2861	1.2868	1.288	C5–C10–C11	121.63	121.26	119.86	C4–C5–C10–N18	–163.31	–163.31	164.15
C11–C12	1.5435	1.5427	1.523	C5–C10–N18	116.24	116.28	117.56	C6–C1–C2–C3	0.002	0.004	–1.60
C11–H25	1.0897	1.0894	0.990	C5–C6–H22	120.71	120.91	121.63	C6–C1–C2–O9	178.67	178.67	178.55
C11–H26	1.0912	1.0915	0.990	C6–C5–C10	119.60	119.78	118.95	C6–C1–O7–C8	170.55	170.55	–178.00
C12–N13	1.4574	1.4620	1.459	C6–C1–O7	128.27	128.27	127.61	C6–C5–C4–H21	–179.7	–179.96	–179.51
C12–H27	1.0902	1.0894	0.990	O7–C8–O9	107.29	106.84	109.45	C6–C5–C10–C11	–164.46	–164.46	159.60
C12–H28	1.0924	1.0911	0.990	O7–C8–H23	109.53	109.56	109.83	C6–C5–C10–N18	15.85	15.85	17.25
N13–C14	1.3815	1.3802	1.370	O7–C8–H24	109.50	109.44	109.81	O7–C1–C2–O9	0.06	0.13	1.40
N13–C17	1.3678	1.3629	1.349	O9–C8–H23	109.38	109.46	109.80	O7–C1–C6–C5	178.42	178.54	179.65
C14–C15	1.3716	1.3713	1.360	O9–C8–H24	109.17	109.18	109.81	O7–C1–C6–H22	–1.73	–1.64	0.59
C14–H29	1.0778	1.0776	0.950	C10–C11–C12	111.71	111.99	111.31	O9–C2–C3–C4	–178.68	–178.61	178.55
C15–N16	1.3751	1.3795	1.377	C10–C11–H25	110.75	110.32	109.42	O9–C2–C3–H20	1.32	1.52	–1.53
C15–H30	1.0790	1.0792	0.950	C10–C11–H26	108.46	108.41	109.42	C10–C5–C6–C1	–179.05	–179.29	–178.45
N16–C17	1.3142	1.3197	1.323	C11–C10–N18	122.12	122.46	122.83	C10–C5–C4–H21	–0.79	–1.78	–1.11
C17–H31	1.0802	1.0798	0.950	C11–C12–N13	112.49	112.05	111.10	C10–C5–C6–H22	1.09	0.90	1.52
N18–O19	1.4067	1.4061	1.409	C12–C11–H25	109.89	109.89	109.42	C5–C4–C3–H20	–179.53	–179.49	178.63
O19–H32	0.9631	0.9647	0.985	C12–C11–H26	108.47	108.56	109.43	C12–N13–C14–C15	176.72	177.67	176.55

The used numbering of atoms is as shown in Figure 3.

**Table 3:** Second-order perturbation theory analysis of Fock matrix in natural bond orbital basis for compound **4**.

Donor (*i*)	Occupancy(e)	Acceptor (*j*)	Occupancy(e)	E(2) ^a^ kcal/mol	E(*j*) – E(*i*) ^b^ (a.u)	F(*i*,*j*) ^c^ (a.u)
π(C_1_–C_6_)	1.70639	π*(C_2_–C_3_)	0.36424	20.27	0.29	0.070
π(C_1_–C_6_)	1.70639	π*(C_4_–C_5_)	0.37847	17.86	0.30	0.067
π(C_2_–C_3_)	1.69340	π*(C_1_–C_6_)	0.33036	18.94	0.30	0.068
π(C_2_–C_3_)	1.69340	π*(C_4_–C_5_)	0.37847	19.07	0.30	0.069
π(C_4_–C_5_)	1.69274	π*(C_1_–C_6_)	0.33036	17.00	0.29	0.063
π(C_4_–C_5_)	1.69274	π*(C_2_–C_3_)	0.36424	17.47	0.28	0.063
π(C_4_–C_5_)	1.69274	π*(C_10_–N_18_)	0.18707	17.62	0.28	0.064
π(C_14_–C_15_)	1.85899	π*(N_16_–C_17_)	0.37926	14.88	0.28	0.061
LP2(O_7_)	1.85977	π*(C_1_–C_6_)	0.33036	25.80	0.36	0.090
LP2(O_9_)	1.85270	π*(C_2_–C_3_)	0.36424	27.07	0.35	0.093
LP1(N_13_)	1.55644	π*(C_14_–C_15_)	0.30606	30.67	0.29	0.087
LP1(N_13_)	1.55644	π*(N_16_–C_17_)	0.37926	46.28	0.28	0.103
LP1(N_18_)	1.95533	σ*(C_10_–C_11_)	0.03261	8.70	0.83	0.076
LP1(O_19_)	1.99160	σ*(C_11_–H_25_)	0.01285	0.67	1.11	0.024
LP2(O_19_)	1.90883	π*(C_10_–N_18_)	0.18707	15.97	0.35	0.068

The used numbering of atoms is as shown in Figure 3. ^a^: E(2) means energy of stabilization interactions; ^b^: Energy difference between donor-to-acceptor, *i* and *j* NBO orbitals; ^c^: F(*i*,*j*) is the Fock matrix element between *i* and *j* NBO orbitals.

**Table 4:** Calculated vibrational wavenumbers, observed FT-IR and FT-Raman frequencies, FT-IR and FT-Raman intensities and their assignments with PED % for compound **4**.

Wavenumber			Intensity		Assignment with PED % (≥10%)
Expt.		Calc.	IR ^a^ (km·mol^–1^)	Raman ^b^ (m^2^·sr^−1^)	
ν_IR_ (cm^–^^1^)	ν_Raman_ (cm^–^^1^)	ν_Scal_ (cm^–^^1^)			
		3830	131.118	7.45	ν (O_19_–H_32_) (100)
3144	3145	3128	1.497	8.37	ν_ring II_ (C–H) (99)
3121	3120	3118	2.054	7.851	ν_ring II_ (C–H) (99)
3114	3118	3103	4.324	10.20	ν_ring II_ (C–H) (99)
-	3073	3096	3.035	6.57	ν_ring II_ (C–H) (99)
3018	-	3056	1.286	5.39	ν_ring I_ (C–H) (99)
3007	3001	3026	2.149	10.90	ν_ring I_ (C–H) (99)
2993	-	3017	3.215	11.70	ν_ring I_ (C–H) (99)
-	2970	2977	9.230	2.62	ν_as_ (CH_2_) Me III(93)
2945	2946	2944	29.934	17.60	ν_as_ (CH_2_) Me I (75) + ν_s_ (CH_2_) Me I (25)
-	-	2934	23.671	11.10	ν_s_ (CH_2_) Me III (98)
-	-	2927	4.531	7.86	ν_as_ (CH_2_) Me II (93)
-	2908	2889	6.873	10.60	ν_s_ (CH_2_) Me II (98)
-	2845	2836	118.557	27.20	ν_s_ (CH_2_) Me I (74) + ν_as_ (CH_2_) Me I (26)
-	1629	1629	14.078	73.10	ν (C_10_–N_18_) (71)
1595	1606	1591	24.766	100.00	ν_ring I_ (CC) (53) + β (CH) Ring I (16)
-	-	1581	3.985	20.60	ν_ring I_ (CC) (58) + β (CH) Ring I (14)
-	-	1560	0.336	14.40	*Sci*(HC) Me I (89)
1503	1516	1504	44.673	2.85	β (CH) Ring II (41) + ν (C_17_–N_16_) (28) +ν (NC) (12)
-	-	1492	179.197	6.28	β (CH) Ring I (50) + ν_ring I_ (CC) (32)
1493	1494	1490	17.736	6.84	ν (C_14_–C_15_) (35) + β (CH) Ring II (29) +ν (NC) (11)
1450	-	1457	14.740	4.35	*Sci* (CH_2_) Me II (37) + τ (CN) (31) + *Sci* (CH_2_) Me III (16)
-	-	1444	3.326	21.40	ω (CH_2_) Me I (88)
1439	1440	1442	25.303	19.50	*Sci* (CH_2_) Me II + τ (CN) (25) + *Sci* (CH_2_) Me III (14)
1428	-	1424	55.376	5.77	ν_ring I_ (CC) (44) + β (CH) Ring I (34)
1401	1403	1390	17.021	13.50	τ (CN) (47) + ω (CH_2_) Me III (30)
1364	-	1369	2.376	5.68	ν (NC) (28) + τ (CN) (17) + ω (CH_2_) Me III (12) + ρ (CH_2_) Me III (12) + *twi* (CH_2_) Me III (12)
1350	1346	1341	2.477	15.10	β (CH) Ring II (39) + ν (C_17_–N_16_) (31) + ν (NC) (15)
1313	1314	1324	38.626	10.90	β (CH) Ring I (40) + ν (CC) (37) + δ_a_ (Ring I) (10)
-	-	1301	90.153	46.50	ν_ring I_ (CC) (32) + β (CH) Ring I (15)
1280	1291	1293	9.747	28.50	β (CH) Ring II (55) + ν (C_17_–N_16_) (16)
-	1271	1276	18.733	10.70	ω (CH_2_) Me II (62)
1255	1254	1251	4.921	3.83	*Twi* (CH_2_) Me II (34) + τ (CN) (24) + ρ (CH_2_) Me III (10) + *Twi* (CH_2_) Me III (10)
-	-	1225	26.089	28.90	ω (CH_2_) Me I (37)
1225	1232	1224	199.125	31.10	β (NOH) (23) + CO (13) + CC (12)
-	-	1222	78.688	30.70	ν (NC) (16) + CN (13) + β (CH) Ring II (13) + *Twi* (CH_2_) Me I (11)
-	-	1208	103.083	19.30	β (NOH) (23) + ν_ring I_ (CC) (16) + ν (CO) (10) + β (CH) Ring I (10)
-	-	1202	120.699	19.90	ν_ring I_ (CC) (30) + ν (C_5_–C_10_) (10) + β (NOH) (10)
1173	1174	1151	4.028	2.22	ν_ring I_ (CC) (25) + β (CH) Ring I (25)
1147	1141	1136	6.792	2.61	ν (NC) (28) + *Twi* (CH_2_) Me I (14) + ν_ring I_ (CC) (14) + β (CH) Ring I (12)
-	-	1124	5.682	2.44	ρ (CH_2_) Me I (56) + τ (CO) (10) + ν_ring I_ (CC) (10)
1108	1107	1111	12.714	7.30	ν (NC) (32) + β (CH) Ring II (25) + ν (C_14_–C_15_) (11)
1085	1083	1097	54.779	15.00	δ (Ring I) (20) + ν (CO) (16) + ν (NC) (14)
-	1050	1079	51.301	13.80	β (CH) Ring II (43) + ν (NC) (32) + ν (C_14_–C_15_)(15)
1035	1030	1032	12.857	19.20	τ (CN) (25) + ρ (CH_2_) Me III (21) + *Twi* (CH_2_) Me III (21)
1016	-	1019	121.778	4.74	ν (OC) (36) + τ (CN) (11)
957	956	1000	50.386	9.32	ν (CC) (26) + ν_ring I_ (CC) (14)
-	-	972	6.193	14.00	ν (CC)(66)
936	-	966	16.460	14.90	δ (Ring I) (34) + ν (NC) (27) + ν (CN) (13)
-	-	935	3.579	3.29	ω (CH) Ring I (86)
924	922	921	100.257	9.39	ν (N_18_–O_19_) (34) + τ (CN) (14) +ρ (CH_2_) Me III (10) + *Twi* (CH_2_) Me III (10)
895	893	896	54.208	6.21	ν (CO) (70) + δ (Ring I) (14)
878	-	883	61.051	8.07	ν (CO) (24) + ν (N_18_–O_19_) (16) + δ (Ring I) (13)
854	850	851	24.434	2.27	ω (CH) Ring I (77)
-	-	841	2.112	2.68	ω (CH) Ring II(87)
-	-	818	7.104	4.86	γ (Ring II) (89)
808	814	809	21.360	7.71	ω (CH) Ring I (84)
799	-	798	13.387	28.60	ν_ring I_ (CC) (33) + ν (CO) (18) + ν (CO) (13)
752	-	785	32.319	4.73	ω (CH) Ring II (77) + ORO (11)
743	-	756	2.956	6.19	τ (CN) (40) + ρ (CH_2_) Me III (17) + *twi* (CH_2_) Me III (17)
-	-	743	2.742	5.72	τ (CN) (26) + ρ (CH_2_) Me III (11) + *twi* (CH_2_) Me III (11)
-	-	733	9.478	12.10	τ (CN) (37) + ρ (CH_2_) Me III (15) + *twi* (CH_2_) Me III (15)
725	721	706	33.336	14.00	ω (CH) Ring II (82) + τ (CN) (14)
716	-	704	3.019	15.40	γ (Ring I) (18) + δ (Ring I) (11) + γ (Ring I) (11)
-	-	690	5.722	5.12	*puc* (Ring I) (61) + ω (CC) (13) + τ_a_ (Ring I) (10)
-	692	650	5.478	2.91	ω (CC) (15) + τ_a_ (Ring I) (12) + τ (Ring I) (10)
658	658	629	17.050	1.48	τ_a_ (Ring II) (69) + τ (CN) (17)
621	626	614	18.672	4.91	δ (Ring II) (17) + ν (C_12_–N_13_) (15) + ν (CC) (11)
578	573	592	11.347	7.42	τ_a_ (Ring II) (62) + ω (CC) (11)
556	-	582	14.299	9.94	δ (Ring I) (30)
492	490	530	5.947	2.11	τ (CN) (20) + ω (CC) (14) + *puc* (14)
-	-	447	5.385	7.04	γ (Ring I) (20) + OC (19) + δ (Ring I) (15)
433	449	440	19.054	7.96	β (CNO) (21) + δ (Ring I) (15) + ν (CC) (10)
425	-	424	86.609	9.89	τ (NO) (72)
417	-	405	13.261	4.70	τ_a_ (Ring I) (36) + *butt* (35) + τ (Ring I) (14)
-	387	361	1.486	7.82	τ (CN) (26) + β (CN) (20) + τ (CN) (10)
-	-	341	1.034	10.40	τ (CN) (30) + *Sci* (13) + OC (12)
-	330	333	0.753	9.09	τ (CN) (67) + β (CN) (11)
-	303	303	4.676	15.80	τ (CN) (38) + τ (CN) (15) + *butt* (11)
-	277	260	1.201	20.60	τ (CN) (69)
-	230	222	0.661	34.50	τ (CN) (51) + ρ (OC) (10)
-	-	215	0.930	29.90	τ_a_ (Ring II) + (24) + τ (Ring I) (24)
-	-	180	0.384	13.60	τ (CN) (73) + β (CC) (10)
-	-	138	0.183	22.60	τ (CN) (37) + *Sci* (20)
-	-	126	7.961	18.10	τ (Ring I) (40) + τ (CN)(18) + τ (CN) (18) + τ (CN) (14)
-	-	91	5.187	119.00	τ (CN) (27) + τ (Ring I) (17) + ω (CC) (12) + τ (CN)(10)
-	-	72	1.075	156.00	τ (CN) (99)
-	-	64	0.692	162.00	τ (CN) (78)
-	-	29	1.601	104.00	τ (CN) (98)
-	-	20	1.176	312.00	τ (CN) (99)
-	-	16	0.169	328.00	τ (CN) (86)

^a^: IR intensity; ^b^: Raman intensity; Ring I: benzodioxole ring; Ring II: imidazole ring; ν: stretching; Me: methylene; ν_s_: symmetric stretching; ν_as_: asymmetric stretching; β: bending; τ: torsion; puc: puckering; ω: wagging; *twi*: twisting; ρ: rocking; *Sci*: scissoring; γ: out-of-plane bending; δ: in-plane bending; *butt*: butterfly mode; τ_a_: out-of-plane torsion.

**Table 5:** Calculated and experimental chemical shift values for compound **4**.

Carbon Atoms (^13^C)			Hydrogen Atoms (^1^H)		
Atoms	Value (ppm)		Atoms	Value (ppm)	
	Calc.	Exp.		Calc.	Exp.
C1	127.25	148.0	H20	5.81	6.84
C2	127.81	148.3	H21	7.01	7.06
C3	89.62	108.5	H22	5.89	7.13
C4	107.84	120.5	H23	5.40	6.04
C5	107.85	130.3	H24	5.20	6.04
C6	89.80	106.1	H25	1.92	3.14
C8	87.78	101.7	H26	2.23	3.14
C10	132.24	153.7	H27	3.40	4.17
C11	29.66	28.2	H28	3.64	4.17
C12	38.54	43.3	H29	6.05	6.89
C14	100.78	119.9	H30	5.94	7.19
C15	109.30	128.3	H31	6.38	7.66
C17	115.68	137.5			

The used numbering of atoms is as shown in Figure 3.

**Table 6:** Antifungal activity of compound **4** and ketoconazole against different *Candida* species and *Aspergillus niger*.

Compound No.	MIC (μmol/L)			
	*C. albicans*	*C. tropicalis*	*C.parapsilosis*	*Aspergillus niger*
**4**	987.43	987.43	987.43	987.43
Ketoconazole	7.53	15.05	15.05	15.05

**Table 7:** The crystallographic data and refinement information.

Chemical Formula	2(C_13_H_13_N_3_O_3_)·C_3_H_8_O
Molecular weight	578.62
Crystal system, space group	Monoclinic, *P*2_1_
Temperature (K)	296
*a*, *b*, *c* (Å)	9.0963(3), 14.7244(6), 10.7035(4)
β(°)	94.298(3)
*V* (Å^3^)	1429.57(9)
*Z*	2
Radiation type	Cu Kα
µ (mm^–1^)	0.81
Crystal size (mm)	0.40 × 0.23 × 0.11
**Data collection**	
Diffractometer	Bruker APEX-II CCD diffractometer
Absorption correction	Multi-scan, SADABS Bruker 2014
T_min_, T_max_	0.740, 0.916
No. of measured, independent and observed [I > 2σ(I)] reflections	6654, 3851, 2788
R_int_	0.039
**Refinement**	
R[*F*^2^> 2σ(*F*^2^)] ^a^, wR(*F*^2^) ^b^, S	0.059, 0.144, 1.03
No. of reflections	3851
No. of parameters	393
No. of restraints	1
H-atom treatment	H atoms treated by a mixture of independent and constrained refinement
Δρ_max_, Δρ_min_ (e·Å^–3^)	0.26, −0.23

^a^: R is the residual factor for the reflections; ^b^: wR is the weighted residual factor for all the reflections.

## Supplementary Materials

Supplementary materials are available online.
