# Supplementary material for: Synthesis, X-ray Single Crystal Structure, Molecular Docking and DFT Computations on N-[(1E)-1-(2H-1,3-Benzodioxol-5-yl)-3-(1H-imidazol-1-yl)propylidene]-hydroxylamine: A New Potential Antifungal Agent Precursor
Source: Molecules. 2017 Feb 28;22(3):373. doi: 10.3390/molecules22030373 (PMC6155236; doi:10.3390/molecules22030373)
Supplement: Supplementary file 1 [file molecules-22-00373-s001.pdf]

Supplementary Material: Synthesis, X-ray Single Crystal Structure, Molecular Docking and DFT Computations on *N*-[(1*E*)-1-(2*H*-1,3-benzodioxol-5-yl)-3-(1*H*-imidazol-1-yl)propylidene]-hydroxylamine: a New Potential Antifungal Agent Precursor

Reem I. Al-Wabli, Alwah R. Al-Ghamdi, Hazem A. Ghabbour, Mohamed H. Al-Agamy, J. Clemy Monickaand, I. Hubert Joe and Mohamed I. Attia*

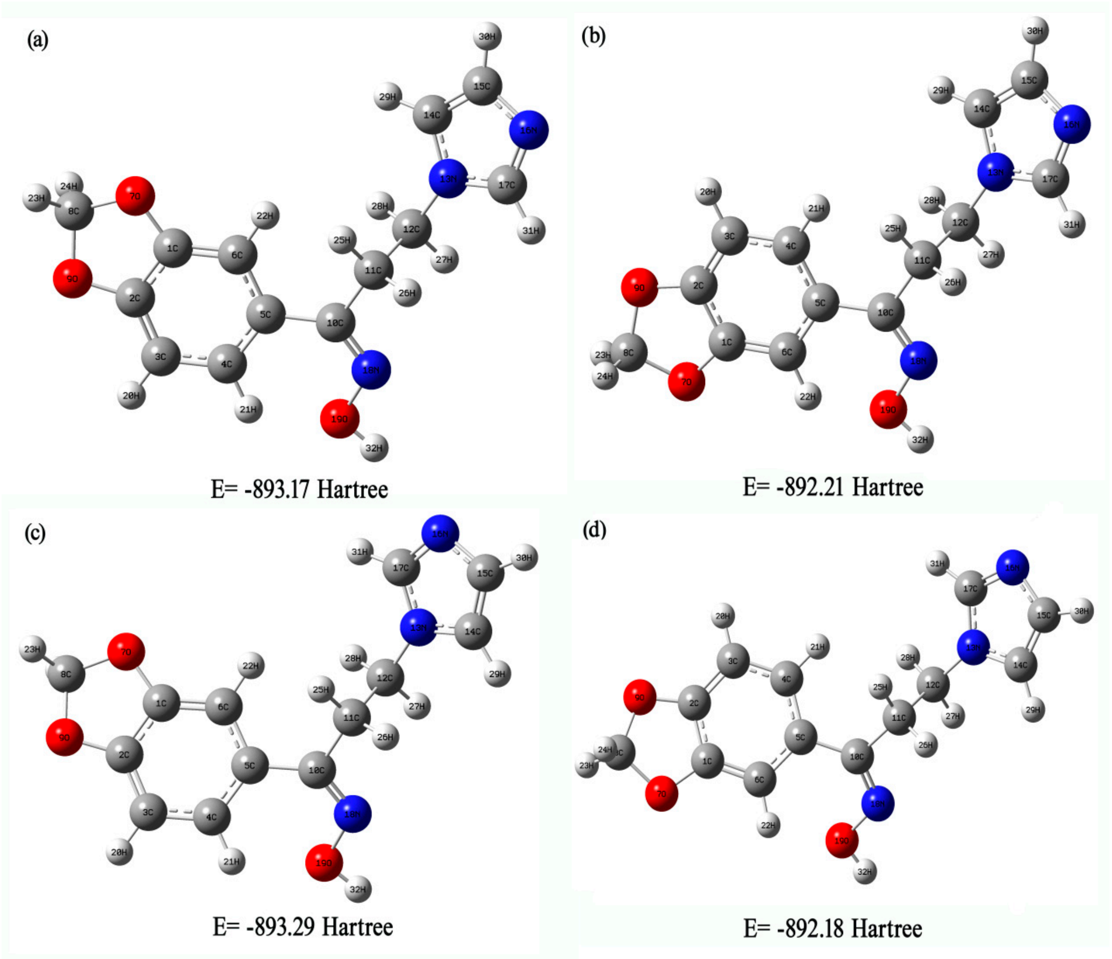

Figure S1: Various conformers of the compound **4** (in solution phase) and with their relative optimum energy

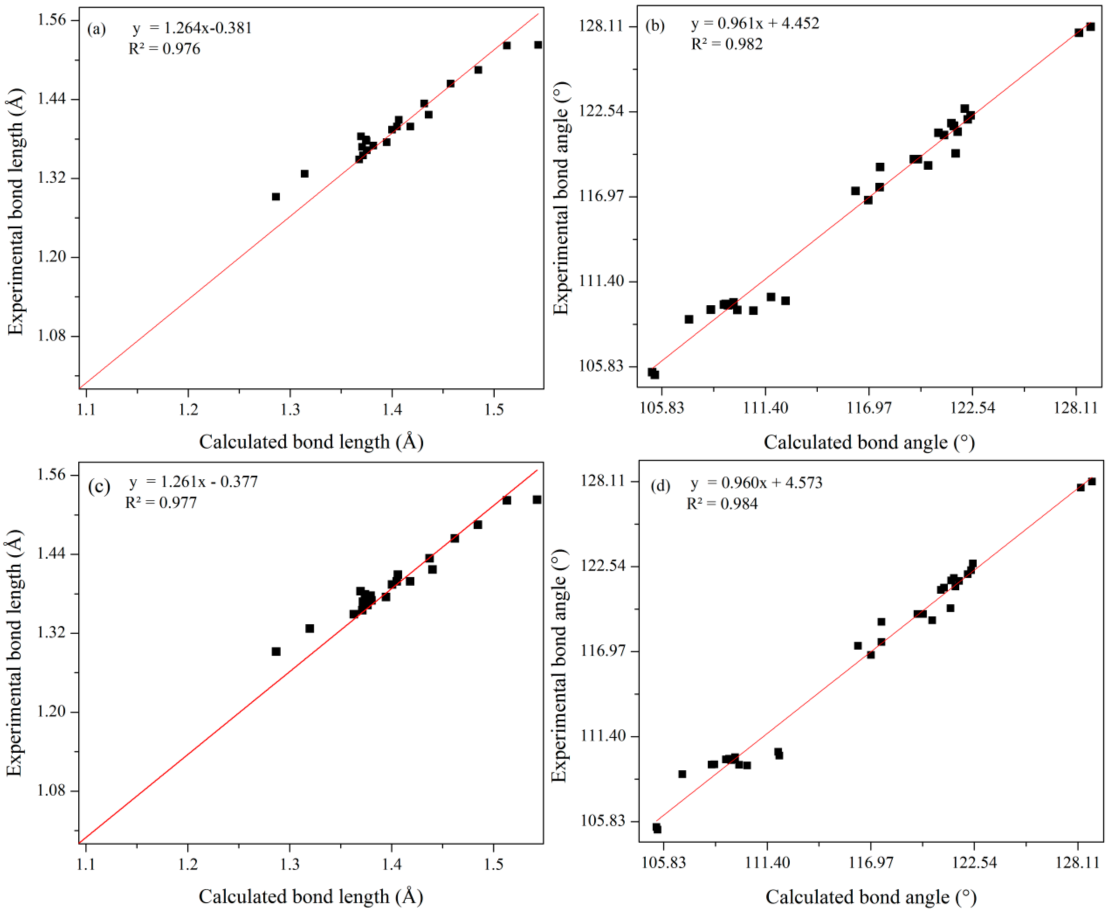

Figure S2: Linear curve fitting plots of calculated and experimental bond lengths and bond angles parameters for compound **4** in the gas phase (a,b) and solution phase (c,d).

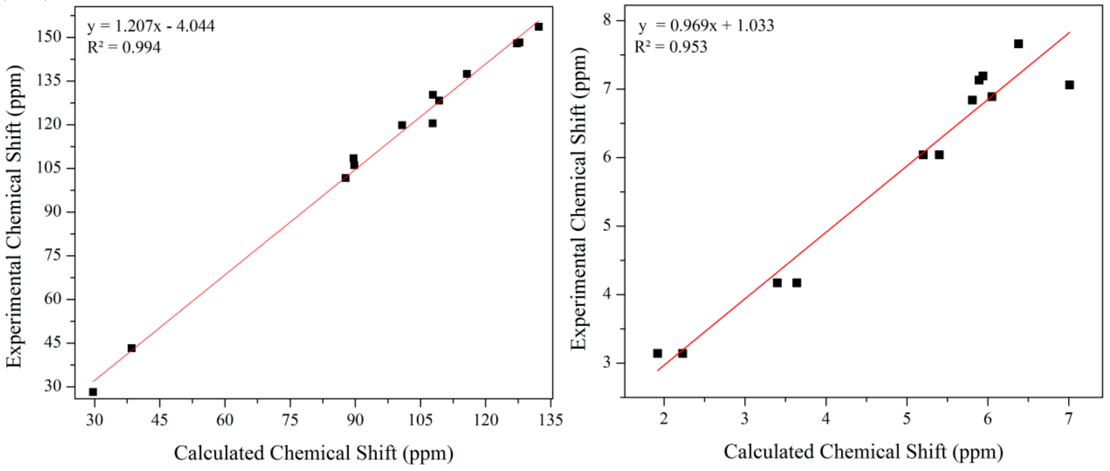

Figure S3: Correlation graphs between the calculated and observed NMR chemical shift values for ^{13}C (left) and ^{1}H (right) of the title oxime **4**.

Table S1: Definition of internal valence coordinates of the title oxime **4**.

No	Symbol	Type	Definition
**Stretching**			
1-6	R_i	C-C (ring)	C_1-C_2, C_2-C_3, C_3-C_4, C_4-C_5, C_5-C_6, C_6-C_1
7-12	r_i	C-H (ring)	C_4-H_{21}, C_6-H_{22}, C_3-H_{20}, C_{14}-H_{29},C_{15}-H_{30},C_{17}-H_{31}
13	R_i	C-C	C_5-C_{10}
14-15	r_i	CO	C_2-O_9, C_1-O_7
16-17	r_i	CO	C_8-O_9, C_8-O_7
18-19	R_i	CC	C_{11}-C_{10}, C_{11}-C_{12}
20-25	r_i	C-H (methylene)	C_8-H_{24}, C_8-H_{23}, C_{11}-H_{25}, C_{11}-H_{26}, C_{12}-H_{28}, C_{12}-H_{27}
26	r_i	CN	C_{12}-N_{13}
27-30	r_i	NC	C_{14}-N_{13},N_{13}-C_{17},C_{17}-N_{16},N_{16}-C_{15}
31	R_i	CC	C_{14}-C_{15}
32	r_i	CN	C_{10}-N_{18}
33	P_i	NO	N_{18}-O_{19}
34	P_i	OH	O_{19}-H_{32}
**Bending**			
35-46	β_i	H-C-C (ring)	H_{20}-C_3-C_2, H_{20}-C_3-C_4, H_{21}-C_4-C_3, H_{21}-C_4-C_5, H_{22}-C_6-C_1, H_{22}-C_6-C_5, H_{29}-C_{14}-N_{13}, H_{29}-C_{14}-C_{15}, H_{30}-C_{15}-C_{14}, H_{30}-C_{15}-N_{16}, H_{31}-C_{17}-N_{16}, H_{31}-C_{17}-N_{13}
47-48	β_i	C-C-C (ring)	C_{10}-C_5-C_6, C_{10}-C_5-C_4
49-54	δ_i	C-C-C (ring)	C_6-C_1-C_2, C_1-C_2-C_3, C_2-C_3-C_4, C_3-C_4-C_5, C_4-C_5-C_6, C_5-C_6-C_1
55	α_i	H-C-H (methylene)	H_{24}-C_8-H_{23}
56	γ_i	O-C-O (methylene)	O_9-C_8-O_7
57-60	β_i	H-C-O (methylene)	H_{24}-C_8-O_7, H_{23}-C_8-O_7, H_{24}-C_8-O_9, H_{23}-C_8-O_9
61	α_i	H-C-H (methylene)	H_{25}-C_{11}-H_{26}
62	γ_i	C-C-C (methylene)	C_{10}-C_{11}-C_{12}
63-66	β_i	H-C-C (methylene)	H_{25}-C_{11}-C_{12}, H_{26}-C_{11}-C_{12}, H_{25}-C_{11}-C_{10}, H_{26}-C_{11}-C_{10}
67	α_i	H-C-H (methylene)	H_{28}-C_{12}-H_{27}
68	γ_i	O-C-O (methylene)	C_{11}-C_{12}-N_{13}
69-72	β_i	H-C-N (methylene)	H_{28}-C_{12}-N_{13}, H_{27}-C_{12}-N_{13}, H_{28}-C_{12}-C_{11}, H_{27}-C_{12}-C_{11}
73-77	δ_i	C-C-O (ring)	C_2-C_1-O_7, C_1-O_7-C_8, O_9-C_2-C_1, O_7-C_8-O_9, C_8-O_9-C_2
78-79	β_i	C-N-C	C_{12}-N_{13}-C_{14}, C_{12}-N_{13}-C_{17}
80-81	β_i	C-C	O_9-C_2-C_1-C_6, O_7-C_1-C_2-C_3
82-86	δ_i	C-C-N (ring)	C_{17}-N_{13}-C_{14}, N_{13}-C_{14}-C_{15}, N_{16}-C_{17}-N_{13}, C_{14}-C_{15}-$N1_6$, C_{15}-N_{16}-C_{17}
87	α_i	C-O-C	C_5-C_{10}-C_{11}
88-89	β_i	N-O-C	N_{18}-C_{10}-C_{11}, N_{18}-C_{10}-C_5
90	α_i	N-O-H	N_{18}-O_{19}-H_{32}

91	α_i	C-N-O	C_{10}-N_{18}-O_{19}
**Wagging**			
92-97	ω_i	H-C-C	H_{20}-C_3-C_2-C_4, H_{21}-C_4-C_3-C_5, H_{22}-C_6-C_1-C_5, H_{29}-C_{14}-N_{13}-C_{15}, H_{30}-C_{15}-C_{14}-N_{16}, H_{31}-C_{17}-N_{16}-N_{13}
98-100	ω_i	C-C-C	C_{10}-C_5-C_6-C_4, C_5-C_{10}-C_{11}-N_{18}, C_{12}-N_{13}-C_{14}-C_{17}
101	ω_i	N-C-C	N_{18}-C_{10}-C_5-C_{11}
**Torsion**			
102-107	τ_i	C-C-C (ring)	C_6-C_1-C_2-C_3, C_1-C_2-C_3-C_4, C_2-C_3-C_4-C_5, C_3-C_4-C_5-C_6, C_4-C_5-C_6-C_1, C_5-C_6-C_1-C_2
108-117	τ_i	C-C-C (ring)	C_2-C_1-O_7-C_8, C_1-O_7-C_8-O_9, O_7-C_8-O_9-C_2, C_8-O_9-C_2-C_1, O_9-C_2-C_1-O_7, C_{17}-N_{13}-C_{14}-C_{15}, N_{13}-C_{14}-C_{15}-N_{16}, C_{14}-C_{15}-N_{16}-C_{17}, C_{15}-N_{16}-C_{17}-N_{13}, N_{16}-C_{17}-N_{13}-C_{14}
118-121	τ_i	C-C	C_4-C_5-C_{10}-N_{18}, C_4-C_5-C_{10}-C_{11}, C_6-C_5-C_{10}-N_{18}, C_6-C_5-C_{10}-C_{11}
122-123	τ_i	C-N	C_5-C_{10}-N_{18}-O_{19}, C_{11}-C_{10}-N_{18}-O_{19}
124	τ_i	N-O	C_{10}-N_{18}-O_{19}-H_{32}
125-126	τ_i	O-C	C_1-O_7-C_8-H_{23}, C_1-O_7-C_8-H_{24}
127-128	τ_i	O-C	C_2-O_9-C_8-H_{23}, C_2-O_9-C_8-H_{24}
129-134	τ_i	C-C	C_5-C_{10}-C_{11}-C_{12}, N_{18}-C_{10}-C_{11}-C_{12}, C_5-C_{10}-C_{11}-H_{25}, C_5-C_{10}-C_{11}-H_{26}, N_{18}-C_{10}-C_{11}-H_{25}, N_{18}-C_{10}-C_{11}-H_{26}
135-143	τ_i	C-C	C_{10}-C_{11}-C_{12}-N_{13}, H_{25}-C_{11}-C_{12}-N_{13}, H_{26}-C_{11}-C_{12}-N_{13}, C_{10}-C_{11}-C_{12}-H_{27}, H_{25}-C_{11}-C_{12}-H_{27}, H_{26}-C_{11}-C_{12}-H_{27}, C_{10}-C_{11}-C_{12}-H_{28}, H_{25}-C_{11}-C_{12}-H_{28}, H_{26}-C_{11}-C_{12}-H_{28}
144-149	τ_i	C-N	C_{11}-C_{12}-N_{13}-C_{17}, C_{11}-C_{12}-N_{13}-C_{14}, H_{27}-C_{12}-N_{13}-C_{17}, H_{27}-C_{12}-N_{13}-C_{14}, H_{28}-C_{12}-N_{13}-C_{17}, H_{28}-C_{12}-N_{13}-C_{14}

Table S2: Definition of local symmetry coordinates and the corresponding force constants (mdyne/Å) with the used scale factors of the title oxime **4**.

No	Symbol	Definition	Scale factors
1-6	ν (CC)	R_1, R_2, R_3, R_4 , R_5, R_6	0.88747
7-12	ν (CH)	r_7, r_8, r_9, r_{10}, r_{11}, r_{12}, r_{13}	0.88747
13	ν (CC)	R_{14}	0.88747
14-15	ν (CO)	r_{15}, r_{16}	0.88747
16-17	ν (CO)	r_{17}, r_{18}	0.88747
18-19	ν (CC)	R_{19}, R_{20}	0.88747
20-21	ν (CN)	R_9, R_{10}	0.88747
22	ν_s (CH_2)	$(r_{21}+r_{22})/\sqrt{2}$	0.88747

23	ν_{as} (CH_2)	$(r_{21}-r_{22})/\sqrt{2}$	0.88747
24	ν_{s} (CH_2)	$(r_{23}+r_{24})/\sqrt{2}$	0.88747
25	ν_{as} (CH_2)	$(r_{23}-r_{24})/\sqrt{2}$	0.88747
26	ν_{s} (CH_2)	$(r_{25}+r_{26})/\sqrt{2}$	0.88747
27	ν_{as} (CH_2)	$(r_{25}-r_{26})/\sqrt{2}$	0.88747
28	ν (CN)	r_{27}	0.91875
29-32	ν (NC)	r_{28}, r_{29}, r_{30}, r_{31}	0.91875
33	ν (CC)	R_{31}	0.88747
34	ν (CN)	r_{32}	0.96675
35	ν (NO)	P_{33}	0.91875
36	ν (OH)	P_{34}	0.99931
**Bending**			
37-39	β (CH)	$(\beta_{35}-\beta_{36})/\sqrt{2}$, $(\beta_{37}-\beta_{38})/\sqrt{2}$, $(\beta_{39}-\beta_{40})/\sqrt{2}$	1.03060
40	β (CC)	$(\beta_{47}-\beta_{48})/\sqrt{2}$	1.03060
41	δ (Ring-I)	$(\delta_{49}-\delta_{50}+\delta_{51}-\delta_{52}+\delta_{53}-\delta_{54})/\sqrt{6}$	1.03060
42	γ(Ring- I)	$(2\delta_{49}-\delta_{50}-\delta_{51}+2\delta_{52}-\delta_{53}-\delta_{54})/\sqrt{6}$	1.03060
43	τ_a (Ring-I)	$(\delta_{50}-\delta_{51}+\delta_{53}-\delta_{54})/2$	1.03060
44	*Sci* (CH_2)	$(5\alpha_{55}+\gamma_{56})/\sqrt{26}$	0.94031
45	*Sci* (CC)	$(\alpha_{55}+5\gamma_{56})/\sqrt{26}$	0.94031
46	ρ (CH_2)	$(\beta_{57}-\beta_{58}+\beta_{59}-\beta_{60})/2$	0.94031
47	ω (CH_2)	$(\beta_{57}+\beta_{58}-\beta_{59}-\beta_{60})/2$	0.94031
48	*Twi* (CH_2)	$(\beta_{57}-\beta_{58}-\beta_{59}+\beta_{60})/2$	0.94031
49	*Sci* (CH_2)	$(5\alpha_{61}+\gamma_{62})/\sqrt{26}$	0.94031
50	*Sci* (CC)	$(\alpha_{61}+5\gamma_{62})/\sqrt{26}$	0.94031
51	ρ (CH_2)	$(\beta_{63}-\beta_{64}+\beta_{65}-\beta_{66})/2$	0.94031
52	ω (CH_2)	$(\beta_{63}+\beta_{64}-\beta_{65}-\beta_{66})/2$	0.94031
53	*Twi* (CH_2)	$(\beta_{63}-\beta_{64}-\beta_{65}+\beta_{66})/2$	0.94031
54	*Sci* (CH_2)	$(5\alpha_{67}+\gamma_{68})/\sqrt{26}$	0.94031
55	*Sci* (CC)	$(\alpha_{67}+5\gamma_{68})/\sqrt{26}$	0.94031
56	ρ (CH_2)	$(\beta_{69}-\beta_{70}+\beta_{71}-\beta_{72})/2$	0.94031
57	ω (CH_2)	$(\beta_{69}+\beta_{70}-\beta_{71}-\beta_{72})/2$	0.94031
58	*Twi* (CH_2)	$(\beta_{69}-\beta_{70}-\beta_{71}+\beta_{72})/2$	0.94031
59	τ (Ring-I)	$(\delta_{73}-0.809(\delta_{74}+\delta_{77})+0.309(\delta_{75}+\delta_{76}))/\sqrt{2.5}$	1.02718
60	δ (Ring-I)	$(-1.118(\delta_{74}-\delta_{77})+1.809(\delta_{75}-\delta_{76}))/\sqrt{4.809}$	1.02718
61-63	β (CH)	$(\beta_{41}-\beta_{42})/\sqrt{2}$, $(\beta_{43}-\beta_{44})/\sqrt{2}$, $(\beta_{45}-\beta_{46})/\sqrt{2}$	1.02718
64	β (CN)	$(\beta_{78}-\beta_{79})/\sqrt{2}$	1.02718
65	*butt*	$(\beta_{80}-\beta_{81})/\sqrt{2}$	1.02718
66	τ (Ring-II)	$(\delta_{82}-0.809(\delta_{83}+\delta_{86})+0.309(\delta_{84}+\delta_{85}))$	0.77357

		/√2.5	
67	δ (Ring-II)	$(-1.118(\delta_{83}-\delta_{86})+1.809(\delta_{84}-\delta_{85}))/\sqrt{4.809}$	0.77357
68	δ (COC)	$(2\alpha_{87}-\alpha_{88}-\alpha_{89})/\sqrt{6}$	0.77357
69	β (NOH)	α_{90}	0.77357
70	β (CNO)	α_{91}	0.77357
**Wagging**			
71-73	ω (CH)	$\omega_{92}, \omega_{93}, \omega_{94}$	0.93936
74-75	ω (OC)	ω_{98}, ω_{99}	0.93936
76	ω (NC)	ω_{101}	0.93936
77-79	ω (CH)	$\omega_{95}, \omega_{96}, \omega_{97}$	0.93936
80	ω (CC)	ω_{100}	0.93936
**Torsion**			
81	*puc* (Ring-I)	$(\tau_{102}-\tau_{103}+\tau_{104}-\tau_{105}+\tau_{106}-\tau_{107})/\sqrt{6}$	0.88191
82	τ (Ring-I)	$(\tau_{102}-\tau_{104}+\tau_{105}-\tau_{107})/2$	0.88191
83	τ_a (Ring-I)	$(-\tau_{102}+2\tau_{103}-\tau_{104}-\tau_{105}+2\tau_{106}-\tau_{107})/\sqrt{12}$	0.88191
84	τ (Ring-II)	$(0.309(\tau_{108}+\tau_{112})-0.809(\tau_{109}+\tau_{111})+\tau_{110})/\sqrt{2.5})$	0.88191
85	τ_a (Ring-II)	$(-1.118(\tau_{111}-\tau_{109})+1.809(\tau_{112-1108}))/\sqrt{4.809}$	0.88191
86	τ (Ring-II)	$(0.309(\tau_{113}+\tau_{117})-0.809(\tau_{114}+\tau_{116})+\tau_{115})/\sqrt{2.5})$	0.88191
87	τ_a (Ring-II)	$(-1.118(\tau_{116}-\tau_{114})+1.809(\tau_{117-113}))/\sqrt{4.809}$	0.88191
88	τ (CC)	$(\tau_{118}+\tau_{119}+\tau_{120}+\tau_{121})/2.5$	0.92067
89	τ (CN)	$(\tau_{122}+\tau_{123})/\sqrt{2}$	0.92067
90	τ (NO)	τ_{124}	0.92067
91	τ (OC)	$(\tau_{125}+\tau_{126})/\sqrt{2}$	0.92067
92	τ (CC)	$(\tau_{127}+\tau_{128})/\sqrt{2}$	0.92067
93	τ (CC)	$(\tau_{129}+\tau_{130}+\tau_{131}+\tau_{132}+\tau_{133}+\tau_{134})/0.166667$	0.92067
94	τ (CC)	$(\tau_{135}+\tau_{136}+\tau_{137}+\tau_{138}+\tau_{139}+\tau_{140}+\tau_{141}+\tau_{142}+\tau_{143})/3$	0.92067
95	τ (CN)	$(\tau_{144}+\tau_{145}+\tau_{146}+\tau_{147}+\tau_{148}+\tau_{149})/0.5$	0.92067
